# Cognitive Emotion Regulation Strategies in Anxiety and Depression Understood as Types of Personality

**DOI:** 10.3389/fpsyg.2018.00856

**Published:** 2018-06-12

**Authors:** Ewa Domaradzka, Małgorzata Fajkowska

**Affiliations:** Institute of Psychology, Polish Academy of Sciences, Warsaw, Poland

**Keywords:** cognitive emotion regulation, personality types, types of anxiety, types of depression, anxiety, depression

## Abstract

The identification of distinctive and overlapping features of anxiety and depression remains an important scientific problem. Currently, the literature does not allow to determine stable similarities and differences in the use of cognitive emotion regulation strategies (CERS) in anxiety and depression, especially concerning the adaptive strategies. Consequently, the aim of this study was to identify the overlapping and distinctive patterns of CERS use in the recently proposed types of anxiety and depression in a general population. In this dimensional approach, types of anxiety and depression are considered as personality types and distinguished based on their specific structural composition and functional role (reactive or regulative) in stimulation processing. 1,632 participants from a representative sample completed the Anxiety and Depression Questionnaire (measuring the Arousal and Apprehension Types of anxiety and the Valence and Anhedonic Types of depression) and the Cognitive Emotion Regulation Questionnaire. Regression analyses were conducted with the affective types as predictors. The co-occurrence of the types was accounted for in order to examine their independent relationships with the CERS. We found that reactive arousal anxiety was not related to any strategies, while regulative apprehension anxiety primarily predicted the use of rumination, which is presumably related to the type's cognitive structural components. The strategy specific to reactive valence depression was other-blame (as predicted by the high negative affect in its structure), and the regulative, most structurally complex anhedonic depression predicted the use of the largest number of strategies, including the adaptive ones. The relationships between the types of depression and self-blame and refocus on planning were moderated by sex but the effects were small. These findings fit into the current trend of exploring the shared and specific features of anxiety and depression, which might facilitate their differentiation by identifying CERS that are characteristic for the specific types. This information can be used for supporting diagnosis and targeting selected strategies in therapy both in clinical and non-clinical populations.

## Introduction

The aim of this study is to determine the specific and common patterns in the use of cognitive emotion regulation strategies in the recently proposed types of anxiety and depression (Fajkowska, 2013, 2015). Emotion regulation, which plays a major role in anxiety and depression, is understood as the processes or activities by which individuals can track, evaluate, and influence the nature, course, and expression of emotions (Gross, 1998; Nolen-Hoeksema, 2012). Both anxiety (often associated with worry; Nitschke et al., 2001) and depression (frequently related to repetitive negative thoughts; Beck, 1967) seem to be strongly connected to cognitive processes. Therefore, in this paper we concentrate on the cognitive side of emotion regulation. Additionally, the cognitive elements form an important part of the structure of the proposed anxiety and depression types (Fajkowska, 2013). As we prove below, the studies based on the existing diagnostic categories do not bring conclusive results concerning the relationship between cognitive emotion regulation strategies (CERS) and anxiety and depression. In order to identify the overlapping and distinctive patterns of CERS use in anxiety and depression, we utilize a typology that to some extent capitalizes on the existing models of emotion (cf. Heller, 1993a,b; Watson, 2000) and groups the affective types based on their structure and function. We expect that referring to the structural and functional characteristics will allow for a more precise differentiation of the anxiety and depression types and to explain the similarities in the area of CERS use occurring among them. Differentiation of anxiety and depression is still an important scientific problem (Eysenck and Fajkowska, 2017) that affects diagnosis and therapy, hence the need for approaches that focus on the overlapping and distinctive features and their underlying causes.

### Cognitive emotion regulation in anxiety and depression

Here, cognitive emotion regulation is understood as “an individual's thoughts after having experienced a negative event” (Garnefski et al., 2002a) and is distinct from related constructs, such as coping, which refers to processes happening over longer periods of time (Gross, 2015) or other types of emotion regulation strategies, such as behavioral ones, that are related to specific actions. Studies show that CERS are related to anxiety and depression (e.g., Aldao et al., 2010). Garnefski et al. (2002a) proposed nine cognitive emotion regulation strategies (see Table 1): self-blame, rumination, catastrophizing, other-blame, acceptance, positive refocusing, refocus on planning, putting into perspective, and positive reappraisal. The first four strategies are considered maladaptive, and the latter five adaptive. However, some studies suggest that acceptance should be treated as maladaptive (Martin and Dahlen, 2005). A meta-analysis revealed that maladaptive strategies are more strongly and consistently connected to psychopathology than adaptive strategies, and that mood-related disorders are more strongly connected to emotion regulation strategies than other disorders (Aldao et al., 2010).

**Table 1:** Definitions of the adaptive and maladaptive Cognitive Emotion Regulation Strategies (Garnefski et al., 2002a).

**Strategy**	**Definition**
**MALADAPTIVE**	
Self-blame	Blaming oneself for the negative event
Rumination	Repetitive thinking about the thoughts and feelings about the event
Catastrophizing	Focusing on how terrible the event was
Other-blame	Blaming others for what happened
**ADAPTIVE**	
Acceptance	Resigning to what happened
Positive refocusing	Directing thoughts to pleasant matters
Refocus on planning	Thinking about actions that can help deal with the negative event
Putting into perspective	Diminishing the meaning of the event
Positive reappraisal	Finding a positive side of the negative event

The strategies most frequently described as being related to anxiety are catastrophizing (Garnefski et al., 2002b; Martin and Dahlen, 2005; Garnefski and Kraaij, 2007; Min et al., 2013), rumination (Garnefski et al., 2002b; Martin and Dahlen, 2005; Garnefski and Kraaij, 2007; D'Avanzato et al., 2013), positive reappraisal (inversely), and self-blame (Garnefski et al., 2002b; Martin and Dahlen, 2005; Garnefski and Kraaij, 2007). However, in a longitudinal study, even though a satisfactory temporal stability of the reported strategies was confirmed, in the retest phase only two out of the four strategies (catastrophizing—positively and positive reappraisal—negatively) were still related to anxiety (Garnefski and Kraaij, 2007).

Most authors agree that depression is related to rumination (Garnefski et al., 2002b, 2004; Martin and Dahlen, 2005; Garnefski and Kraaij, 2007; Joormann and Gotlib, 2010; Van Loey et al., 2014), catastrophizing (Garnefski et al., 2002b; Martin and Dahlen, 2005; Garnefski and Kraaij, 2007; Min et al., 2013), and less use of positive reappraisal (Garnefski et al., 2002b; Martin and Dahlen, 2005; Garnefski and Kraaij, 2007; Joormann and Gotlib, 2010; Wang et al., 2014). A few studies pointed to acceptance as being positively connected to depression, in addition to the three already mentioned strategies (Kraaij et al., 2002; Martin and Dahlen, 2005). However, when negative life events and prior depression were controlled for, only acceptance and positive reappraisal remained significant (Kraaij et al., 2002). Moreover, some studies suggest that self-blame (Garnefski et al., 2002b, 2004; Martin and Dahlen, 2005; Garnefski and Kraaij, 2007), other-blame (Garnefski et al., 2002a), positive refocusing (Min et al., 2013; Van Loey et al., 2014; Wang et al., 2014), and refocus on planning (Garnefski et al., 2004; Min et al., 2013) also play a role in depression (self- and other-blame positively; positive refocusing and refocus on planning—negatively).

Apparently, the pattern of the relationship between CERS and anxiety and depression does not allow for a clear differentiation of these phenomena, as most data suggests an overlap in the reported strategies. One study showed that six out of nine strategies correlated with both anxiety and depression, including at a retest after over a year (Garnefski et al., 2002a). Especially the adaptive strategies need to be considered, given that they are often addressed in therapy. Their role in psychopathology is not very clear, but some studies suggest that anxiety and depression are related to a less frequent use of adaptive strategies (Aldao and Nolen-Hoeksema, 2012; Martins et al., 2016); others did not find such a relationship (Nolen-Hoeksema and Aldao, 2011). Moreover, the results are often related to the type of sample that was studied (clinical or non-clinical). For example, Joormann and Gotlib (2010) found that currently depressed participants used more rumination and less reappraisal than remitted and controls, and remitted participants used more rumination than the control group. No difference was found between currently depressed and remitted participants in the use of pondering (a subtype of rumination). Additionally, rumination correlated with depression scores only in the depressed and remitted participants.

We decided to use an alternative approach to anxiety and depression for a few reasons. A review of the vast literature might suggest that the cognitive emotion regulation strategies are transdiagnostic processes, related to a variety of psychopathological units. However, we suggest that the most commonly used diagnostic classifications (DSM-5, American Psychiatric Association, 2013; Spielberger, 1983; Watson, 2000) which are mostly categorical and face the problem of symptom heterogeneity (Gross and Jazaieri, 2014) do not allow for clear patterns of CERS use in anxiety and depression to be found or to explain the similarities and differences between them in the area of emotion regulation. Many studies do not compare anxiety and depression but deal with just one of these phenomena (Kraaij et al., 2002; Garnefski et al., 2004; Joormann and Gotlib, 2010; Min et al., 2013; Lei et al., 2014). Additionally, such an approach does not allow for the co-occurrence to be accounted for; as a result, the specific links might be easily overlooked or misinterpreted. Moreover, even though many studies do not refer to the mechanisms potentially linking anxiety and depression with the CERS, some of them point to the role of cognitive control, goal activation, as well as neural mechanisms responsible for emotional responding, language, and attentional control, among others (McRae et al., 2008; Ochsner and Gross, 2008; Gross et al., 2011). Taking the above into account, we decided to use an approach that distinguishes types of anxiety and depression, allowing to form expectations and interpretations based on the proposed structure and functions of the affective types.

### Types of anxiety and depression

Fajkowska (2013, 2015) proposes that anxiety and depression should be treated as personality types, distinguished based on two criteria: structural complexity and functional role—reactive or regulative—in stimulation processing. Stimulation processing is understood as the transformation of arousal and activation resulting from the incoming stimulation, e.g., sensory, and causing changes in various systems of the organism, e.g., affective or cognitive (Fajkowska, 2017). Both functions are related to individual differences in energy expenditure in a particular time range. The reactive function reflects individual differences in the reception of stimulation, high vigilance to stimuli, and rather automatic readiness to activity (Fajkowska, 2017). An example is anxiety, in which the reactive function can be identified by hypervigilance to threat (Mogg et al., 2000). The regulative function, on the other hand, is connected to more strategic reactions to flowing stimulation. The creative and innovative strategies used to pursue goals in openness (DeYoung, 2010) can serve as an example. Both functions can be identified in one trait; however, one is usually dominant.

#### Types of anxiety

**Figure 1:**
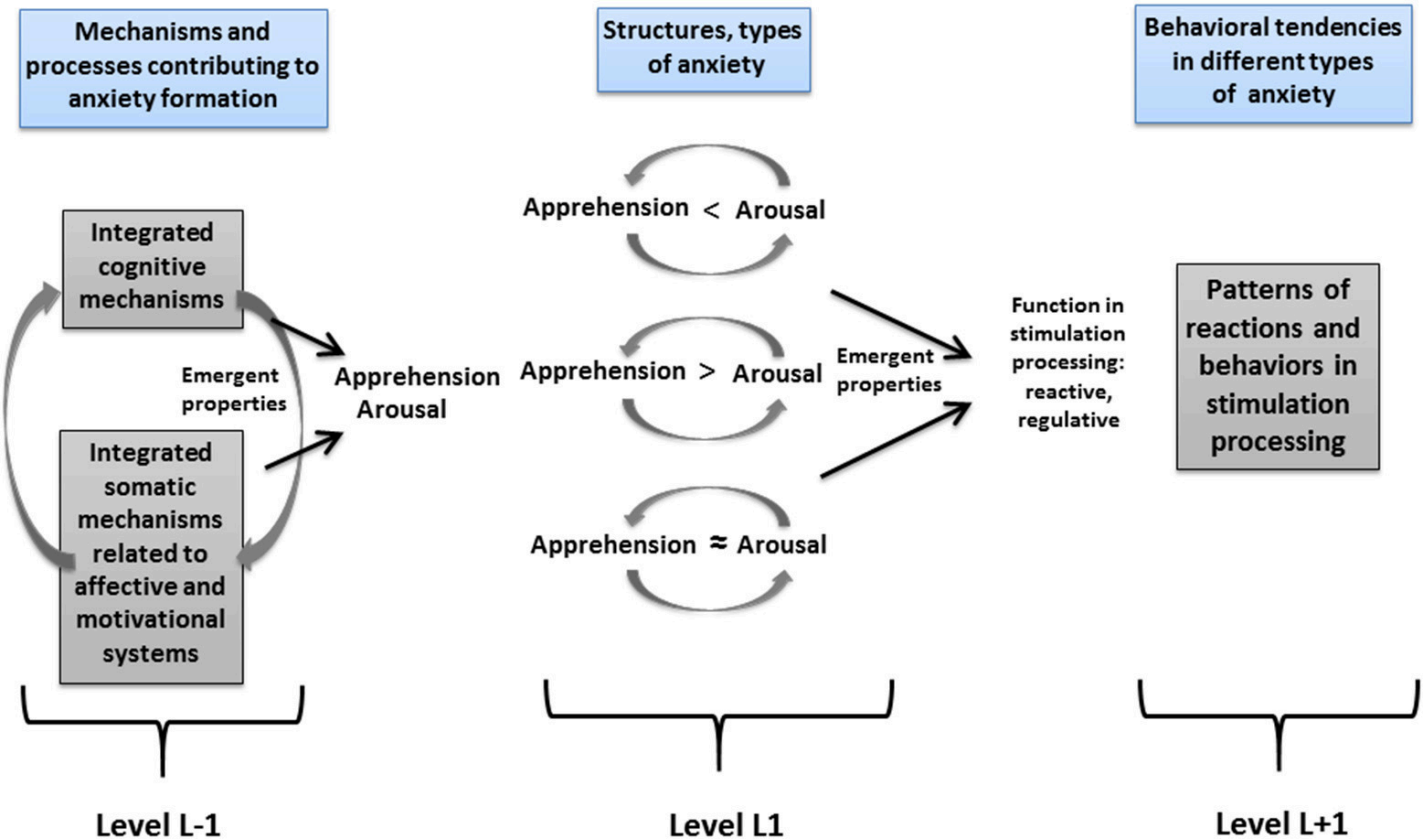
The organization of anxiety types according to the three-level compositional hierarchy. Adapted from Fajkowska (2013), p. 107. Copyright by Eliot Werner, Clinton Corners, NY. Reprinted with permission.

According to Fajkowska (2013, 2015), somatic-related arousal and cognitive-related apprehension are crucial in the formation of anxiety types (see Figure 1). Arousal anxiety is related to physiological hyperarousal and somatic tension (cf. Watson, 2000), while worry is characteristic for apprehension anxiety (Barlow, 1991; Heller, 1993a,b). Arousal anxiety encompasses such diagnostic categories as panic attacks, phobias, or state anxiety (Heller and Nitschke, 1998; Watson, 2000), while apprehension anxiety—Generalized Anxiety Disorder or self-reported trait anxiety (Heller and Nitschke, 1998; DSM-5, American Psychiatric Association, 2013). It is proposed that arousal anxiety is built of: somatic reactivity, panic/phobia, and attentional vigilance/avoidance. Apprehension anxiety consists of: worrisome thoughts, attentional control, and somatic reactivity (see Table 2). The reactive function can be identified as dominant in arousal anxiety (because of increased autonomic reactivity and more automatic stimulation processing—the attentional vigilance-avoidance pattern), while the apprehension type is more related to the regulative function, due to a more strategic (but usually ineffective) stimulation processing pattern, connected with reduced attentional control (Fajkowska, 2013, 2015).

**Table 2:** Structural composition of the anxiety and depression types and their functions in stimulation processing (Fajkowska et al., 2018).

**Type**	**Structural composition**		**Function in stimulation processing**
Arousal anxiety	Somatic reactivity	Elevated autonomic reactivity, psychophysiological arousal, and somatic tension—e.g., trembling hands, heart pounding—resulting from the occurrence of negative and threatening stimuli	Reactive
	Panic/phobia	Panic symptoms, distress, phobias	
	Attentional vigilance/avoidance	“Early” vigilance to threat, usually in the clinical form of anxiety, and “late” attentional avoidance of threat, usually in the non-clinical form	
Apprehension anxiety	Worrisome thoughts	Concerning physical, emotional or symbolic threat to the self; connected with the social appraisal of one's behavior or competence, real or anticipated physical threat, or general problems of the world	Regulative
	Attentional control	Problems in attention switching and concentration, inability to disengage attention from negative experiences, giving in to distracting thoughts, impaired inhibition, especially in processing negative emotional material connected with failure or a negative event	
	Somatic reactivity	Elevated reactivity of the autonomous nervous system while facing threat, or as a result of worrisome thoughts	
Valence depression	Negative affect	Elevated level of anxiety, tension, hostility, anger, sadness, high sensitivity to the self, and social avoidance	Reactive
	Attentional avoidance	Insensitivity to the valence of the emotional material and insensitivity to social stimuli	
Anhedonic depression	Emotional-motivational deficits	Inability to experience pleasure and a lowered reactivity to pleasurable events, difficulties in goal pursuit and taking up activity in order to attain them, inability to attain pleasure or reward oneself by appetitive behaviors	Regulative
	Positive affect	Very low level of positive feelings, such as self-confidence, happiness, or hope	
	Negative affect	Very high level of negative feelings and emotions, such as sadness, guilt, disappointment or anxiety	
	Attentional control	Inability to sustain attention on emotional material, slower and inaccurate reactions to emotional material, lowered ability to sustain effort in processing emotional material regardless of its valence, problems with concentration of attention	

#### Types of depression

**Figure 2:**
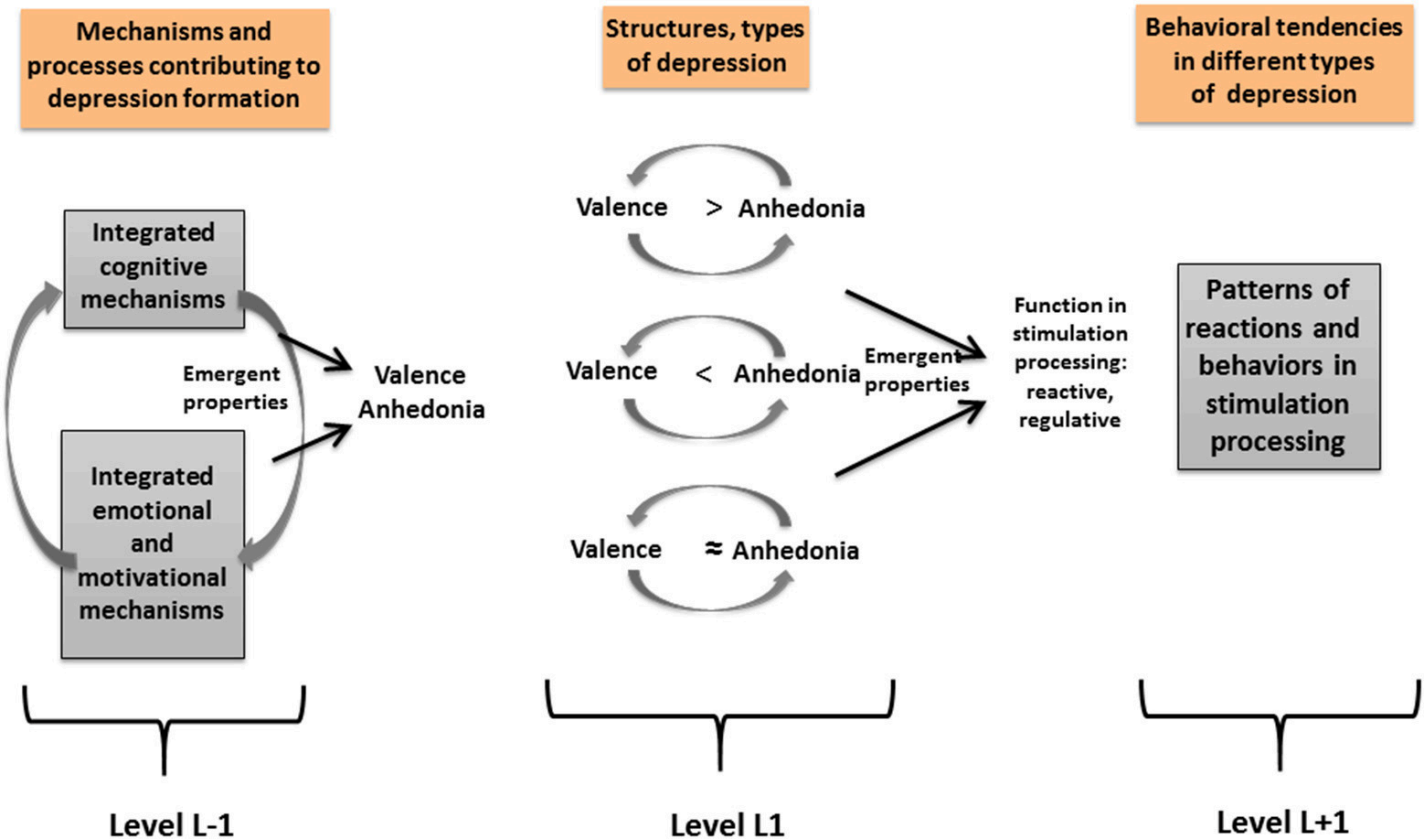
The organization of depression types according to the three-level compositional hierarchy. Adapted from Fajkowska (2013), p. 118. Copyright by Eliot Werner, Clinton Corners, NY. Reprinted with permission.

Further, Fajkowska (2013, 2015) claims that cognitive-related valence insensitivity and emotion- and motivation-related anhedonia lead to the formation of the valence and anhedonic depression, respectively (see Figure 2). Valence insensitivity is characteristic for the non-melancholic types of depression, and anhedonia is typical for the melancholic depression (Heller and Nitschke, 1998; Watson, 2000). Therefore, the Valence Type includes the non-melancholic forms of depression, while the Anhedonic Type encompasses the melancholic forms, described in the DSM-5 (American Psychiatric Association, 2013). It is proposed that valence depression is composed of negative affect and attentional avoidance. Anhedonic depression, on the other hand, includes: emotional-motivational deficits, positive affect, negative affect, and attentional control (see Table 2). Fajkowska (2013, 2015) assumes that valence depression is connected with the dominance of the reactive function, since it is related to more automatic stimulation processing, resulting from attentional avoidance. The dominance of the regulative function can be identified in anhedonic depression because of reduced attentional control and problems with sustaining attention that form a more strategic - but usually ineffective - pattern of stimulation processing.

This categorization (Fajkowska, 2013, 2015) to some extent benefits from existing models of emotion (cf. Heller, 1993a,b; Watson, 2000). However, some notable differences need to be underlined. First of all, anxiety and depression are treated as personality types—stable personality characteristics that can be observed in the general population, rather than as nosological or clinical units. Moreover, they have a complex, dimensional structure. Additionally, this structure determines the functional role (reactive or regulative) of anxiety and depression in stimulation processing. These three assumptions: (1) treating anxiety and depression as dimensional personality types characterized by (2) structural complexity and (3) functional role in stimulation processing summarize the main differences in comparison to the existing models of anxiety and depression. Since Fajkowska bases her theory on the vast literature on the topic, some overlap is also present. For example, the arousal and apprehension types of anxiety (Watson, 2000; Sharp et al., 2015) or the anhedonic depression (Watson, 2000) have been described before; however, these models did not include the structural and functional characteristics of these phenomena. At the same time, within the DSM categorization the attempts to empirically confirm the various depression subtypes have not been successful so far (van Loo et al., 2012). The presented model does not rely on the DSM criteria, though. Additionally, this categorization encompasses anxiety and depression types in one model, therefore allowing for the exploration of the similarities and differences “within” (e.g., anhedonic and valence depression) and “across” (e.g., valence depression and arousal anxiety) types, while taking into account their shared and specific mechanisms, structures, and functions, as well as co-occurrence. Furthermore, this dimensional approach assumes that when high levels of the two types of anxiety or depression are concurrently present, mixed types occur.

### Predictions

Considering the possibility of “grouping” the types of anxiety and depression according to their functional role in processing stimulation (see Table 2), we hypothesized that the use of cognitive emotion regulation strategies will be related to these functional characteristics of the types. Therefore, we expect similarities “across” (between reactive arousal anxiety and valence depression or regulative apprehension anxiety and anhedonic depression) and differences “within” the type pairs of depression or anxiety. For example, both regulative types are connected to reduced attentional control, which might be related to a less frequent use of positive refocusing. Previously, for the purpose of the Anxiety and Depression Questionnaire validation (Fajkowska et al., 2018), we assessed the zero-order correlations between the anxiety and depression types grouped by their functional role and the aggregated adaptive and maladaptive strategies. We found that the regulative types were more strongly correlated with the use of both adaptive and maladaptive strategies than the reactive types. Here, we extend these analyses by including the specific relationships between the types and the strategies, relating them to the structural composition of the types, and controlling the co-occurrence. Hence, we expect that the stronger relationship of the regulative types with the strategies use will hold after controlling for the variance associated with the remaining variables.

We also hypothesized that the use of CERS will be related to the structural composition of the affective types. The domination of the cognitive component in apprehension anxiety (especially worrisome thoughts and reduced attentional control) may be related to more frequent use of rumination and less of positive refocusing. We expect that anhedonic depression will be connected with more frequent use of self-blame, rumination, and catastrophizing, as it is by definition characterized by reduced attentional control, very low positive affect, and high negative affect. Valence depression should be related to a more frequent use of other-blame, since it is connected to a high negative affect, manifesting itself in hostility, tension, anger, anxiety, and sensitivity to the “self.” Moreover, all types of anxiety and depression should be related to more frequent use of non-adaptive strategies (Aldao et al., 2010) and less frequent use of adaptive strategies, especially of positive reappraisal (Garnefski et al., 2002b, 2004; Kraaij et al., 2002; Martin and Dahlen, 2005; Garnefski and Kraaij, 2007; Joormann and Gotlib, 2010).

In accord with the presented theoretical framework of affective types, these types are located at a lower level of the personality system than the behavioral markers (e.g., strategies, placed at the highest level of personality; Fajkowska, 2013) that are connected with and stem from them. This justifies treating affective types as predictors of behavioral acts (i.e., CERS).

Sex differences in the use of CERS are rarely considered or show weak effects (Garnefski and Kraaij, 2006). Nevertheless, women tend to use more cognitive emotion regulation strategies than men in general (Nolen-Hoeksema and Aldao, 2011). Other studies report that women use rumination, positive refocusing, catastrophizing, reappraisal, and acceptance more and self- and other-blame less than men do (Garnefski et al., 2004; Nolen-Hoeksema and Aldao, 2011; Martins et al., 2016). These results are inconsistent, though, apart from rumination, which appears in most empirical reports. Moreover, the same strategies (catastrophizing, rumination, and self-blame) predict depression in both sexes, except for refocus on planning, which predicts depression only in men (Garnefski et al., 2004). Therefore, we hypothesized that women will use more strategies in general, especially rumination. However, we did not make any specific predictions about how gender may modify the relationship between anxiety and depression and CERS use.

## Materials and methods

### Participants

One thousand six hundred and thirty-two participants (52% females) aged 18–65 (*M* = 39, *SD* = 13) from a general population were recruited from an online research panel. The sample matched the demographic structure of the general population. Participation was rewarded with points that were exchangeable for gifts. Since previous studies reported *R*^2^ for the relationships between anxiety/depression and CERS ranging from 0.28 to 0.47 (Garnefski et al., 2004; Garnefski and Kraaij, 2007), we expected medium to large effect sizes. Analysis in G^*^Power 3 (Faul et al., 2007) indicated that detecting a 0.15 effect size with α = 0.05, and power of 0.95 would require a sample size of *N* = 194. Nevertheless, since this was a part of a larger study, we used the whole sample available for the analyses.

### Procedure and materials

This study was carried out in accordance with the recommendations of the Ethics Committee of the Institute of Psychology, Polish Academy of Sciences. The protocol was approved by the Ethics Committee of the Institute of Psychology, Polish Academy of Sciences. After providing informed consent, participants filled out the Anxiety and Depression Questionnaire (Fajkowska et al., 2018) and the Cognitive Emotion Regulation Questionnaire (CERQ, Garnefski et al., 2002a; Marszał-Wiśniewska and Fajkowska, 2010), as well as several other questionnaires that are not described here. Results of the analyses concerning some of the other used measures are described elsewhere (Fajkowska et al., 2017, 2018). The Arousal and Apprehension Anxiety scales were included in one block, and the CERQ and Anhedonic and Valence Depression scales in another block. The second block was filled out 3–4 days after the first one. The order of blocks and questionnaire presentation within them was random.

#### Anxiety and depression questionnaire (ADQ)

The ADQ (Fajkowska et al., 2018) is a self-report questionnaire for the assessment of the anxiety and depression types. It consists of four scales, each directly assessing one type of anxiety or depression, and indirectly measuring the mixed types of anxiety and depression (Fajkowska et al., 2017, 2018) with an “Agree/Disagree” response format. The scales and their subscales, along with sample items, are listed below.

Arousal Anxiety — (45 items, including 4 fillers):

- Somatic Reactivity (22 items): e.g., When I am scared, I feel pain in my chest.
- Panic/Phobia (14 items): e.g., I often get sudden anxiety attacks.
- Attentional Vigilance/Avoidance (5 items): e.g., When I notice a potential threat, I automatically withdraw from the given situation.

Apprehension Anxiety — (48 items):

- Worrisome Thoughts (14 items): e.g., When I start to worry, I cannot stop.
- Attentional Control (23 items): e.g., I cannot concentrate on a difficult task if there are noises around.
- Somatic Reactivity (11 items): e.g., My body reacts intensively to sudden stress.

Valence Depression — (40 items, including 4 fillers):

- Negative Affect (21 items): e.g., I am very often tense.
- Attentional Avoidance (15 items): e.g., It is difficult for me to notice anger in others.

Anhedonic Depression — (64 items):

- Emotional-Motivational Deficits (31 items): e.g., I feel completely bored.
- Positive Affect (13 items): e.g., I often smile honestly and joke.
- Negative Affect (12 items): e.g., I feel worthless.
- Attentional Control (8 items): e.g., Emotional events distract me so much that I later have trouble concentrating.

Empirical data collected during the construction and validation stages provide evidence that the ADQ is a reliable and valid self-rating measure of the anxiety and depression types (Fajkowska et al., 2017, 2018).

#### Cognitive emotion regulation questionnaire

The Cognitive Emotion Regulation Questionnaire (Garnefski et al., 2002a; Marszał-Wiśniewska and Fajkowska, 2010) was designed to measure the cognitive emotion regulation strategies used by individuals after the occurrence of a negative event. It consists of 36 items in 9 scales: self-blame, rumination, catastrophizing, other-blame, acceptance, positive refocusing, refocus on planning, putting into perspective, and positive reappraisal. The participants are asked to mark how often they feel or think in a given way on a 5-point scale, ranging from 1—(almost) never to 5—(almost) always.

## Analyses and results

In order to filter out those who “clicked through” the questionnaires, participants with extremely low variance of raw scores on any of the questionnaires (*N* = 286) were removed. We calculated the number of “I disagree” answers for each ADQ scale (except for Valence Depression as it has no reversed items so a maximum or minimum score is possible even for genuinely responding participants) on the raw scores (before converting the reversed items) and removed participants who provided identical answers to all the items in the questionnaire. Similarly, we removed the participants who had zero variance in their CERQ answers. The means, standard deviations, and Cronbach's alphas of the ADQ and CERQ are presented in Tables 3, 4, respectively.

**Table 3:** Means, standard deviations, and Cronbach's Alphas of the Anxiety and Depression Questionnaire (*N* = 1,346).

	***M***	***SD***	**Cronbach's Alpha**
Arousal anxiety	15.00	10.71	0.94
Apprehension anxiety	23.24	12.77	0.95
Valence depression	10.82	8.80	0.93
Anhedonic depression	18.08	16.07	0.97

**Table 4:** Means, standard deviations, and Cronbach's Alphas of the Cognitive Emotion Regulation Questionnaire in the total sample and in the women and men subgroups.

	**Total sample (*****N*** = **1,346)**		**Women (*****n*** = **719)**		**Men (*****n*** = **627)**		**Cronbach's Alpha**
	***M***	***SD***	***M***	***SD***	***M***	***SD***	
Self-Blame	11.26	2.64	11.23	2.82	11.29	2.43	0.70
Acceptance	13.07	2.31	13.24	2.29	12.86	2.33	0.61
Rumination	12.22	2.70	12.46	2.80	11.94	2.56	0.75
Positive refocusing	12.89	2.55	12.98	2.57	12.78	2.53	0.73
Refocus on planning	14.13	2.47	14.20	2.46	14.05	2.47	0.76
Positive reappraisal	13.72	2.72	13.79	2.72	13.65	2.71	0.78
Putting into perspective	13.05	2.46	13.24	2.51	12.84	2.39	0.70
Catastrophizing	10.75	3.02	10.81	3.11	10.67	2.92	0.78
Other-blame	10.56	2.80	10.36	2.89	10.78	2.65	0.80
All strategies (mean)	12.40	1.41	12.48	1.45	12.32	1.36	0.85

Independent samples *t*-tests were utilized to explore gender differences in the use of CERS. Because of the exploratory character of this analysis, multiple comparisons correction was applied with a significance level of 0.005. The analyses revealed that women use the following strategies more frequently than men: acceptance [*t*_(1344)_ = 3.020, *p* = 0.003, *d* = 0.16], rumination [*t*_(1341)_ = 3.514, *p* < 0.001, *d* = 0.19], and putting into perspective [*t*_(1344)_ = 2.973, *p* = 0.003, *d* = 0.16]. The hypothesis concerning more frequent use of CERS in general in women was not confirmed.

Subsequently, nine hierarchical regression analyses were run. In each of them a different CERS was the predicted variable, and the types of anxiety and depression, age, sex, and the remaining eight CERS were entered as predictors in the first step. In order to assess sex differences, in the second step the interaction between types and sex were entered as predictors. A dummy variable was created where men were coded as 0 and women as 1. The variables were centered at their means for the interaction analyses. Similar analyses were run for the aggregated adaptive and maladaptive strategies.

Table 5 presents the regression coefficients for each of the dependent variables (CERS). Table 6 shows the regression coefficients for the aggregated adaptive and maladaptive strategies. The status of acceptance is unclear (Martin and Dahlen, 2005), therefore it was not included in the aggregated means. We used *p* < 0.05 as the significance level for testing the hypotheses. However, while interpreting the effects that were not related to our predictions we applied the Bonferroni correction for multiple comparisons and used a *p* < 0.001 significance level. Since the types of anxiety and depression are our main focus in this paper, we organized the results section by grouping the results by the affective types. In addition, we report the coefficients for the mutual relationships among the CERS (see Table 5), but we will not discuss them as this is out of this paper's scope. All the regression models were significant. However, the *F* change between the first and second step was significant only in case of self-blame and refocus on planning, indicating that sex moderated the relationships. The change in *R*^2^ was very small, though. Therefore, in the remaining seven models the results from the first step are discussed. In the analyses of the aggregated adaptive and maladaptive strategies use none of the interactions were significant, therefore the results of the first step are reported. Age did not turn out to be a significant predictor of any of the CERS.

**Table 5:** Results of hierarchical regression analyses (standardized Betas) for each of the predicted cognitive emotion regulation strategies (CERS) with types of anxiety and depression, age, sex, and the remaining CERS as predictors (*N* = 1,346).

	**Self-blame**	**Acceptance**	**Rumination**	**Positive refocusing**	**Refocus on planning**	**Positive reappraisal**	**Putting into perspective**	**Catastrophizing**	**Other-blame**
**STEP 1**									
Arousal anxiety	0.070^*^	−0.033	−0.056	0.068	−0.037	−0.014	0.024	−0.008	0.085^*^
Apprehension anxiety	−0.039	−0.004	0.170^***^	−0.112^**^	0.065	−0.042	−0.009	0.037	−0.098^*^
Valence depression	0.104^**^	−0.047	−0.031	0.082^*^	−0.159^***^	0.037	0.038	0.028	0.201^***^
Anhedonic depression	0.156^***^	0.093^*^	0.075^*^	−0.164^***^	0.073^*^	−0.171^***^	−0.127^***^	0.121^***^	−0.089^*^
Age	0.000	0.037	−0.026	0.016	0.037	−0.015	0.032	0.038^*^	−0.048^*^
Sex	−0.078^***^	0.045	0.064^**^	0.021	−0.016	−0.011	0.049^*^	0.024	−0.112^***^
Self–blame		0.205^***^	0.246^***^	−0.143^***^	0.086^**^	0.019	0.065^*^	0.197^***^	−0.196^***^
Acceptance	0.171^***^		0.199^***^	0.080^**^	0.144^***^	0.018	0.141^***^	−0.012	0.014
Rumination	0.292^***^	0.283^***^		−0.063	0.198^***^	0.018	−0.016	0.300^***^	0.175^***^
Positive refocusing	−0.115^***^	0.078^**^	−0.043		0.142^***^	0.139^***^	0.122^***^	0.042	0.060^*^
Refocus on planning	0.090^**^	0.181^***^	0.175^***^	0.185^***^		0.351^***^	0.007	−0.128^***^	0.029
Positive reappraisal	0.026	0.029	0.021	0.233^***^	0.453^***^		0.490^***^	−0.117^***^	0.004
Putting into perspective	0.062^*^	0.161^***^	−0.013	0.144^***^	0.007	0.346^***^		0.025	0.009
Catastrophizing	0.229^***^	−0.017	0.294^***^	0.061	−0.143^***^	−0.100^***^	0.030		0.473^***^
Other-blame	−0.155^***^	0.013	0.117^***^	0.059^*^	0.022	0.002	0.007	0.321^***^
*R*^2^	0.459	0.351	0.543	0.327	0.488	0.599	0.432	0.535	0.315
Model parameters	*F*_(14, 1331)_ = 82.549	*F*_(14, 1331)_ = 52.936	*F*_(14, 1331)_ = 115.229	*F*_(14, 1331)_ = 47.742	*F*_(14, 1331)_ = 90.652	*F*_(14, 1331)_ = 144.629	*F*_(14, 1331)_ = 74.130	*F*_(14, 1331)_ = 111.410	*F*_(14, 1331)_ = 45.166
**STEP 2**									
Arousal anxiety	0.057	−0.052	−0.059	0.114^*^	−0.050	0.003	0.025	−0.033	0.086
Apprehension anxiety	−0.063	0.030	0.183^***^	−0.117^*^	0.079	−0.047	−0.020	0.042	−0.109^*^
Valence depression	0.026	−0.052	0.020	0.063	−0.135^**^	0.021	0.047	0.031	0.121^*^
Anhedonic depression	0.233^***^	0.116^*^	0.009	−0.113^*^	−0.012	−0.162^***^	−0.080	0.112^*^	−0.016
Age	0.000	0.038	−0.027	0.020	0.032	−0.014	0.035	0.036	−0.047^*^
Sex	−0.077^***^	0.045	0.064^**^	0.019	−0.016	−0.012	0.049^*^	0.025	−0.112^***^
Self-blame		0.206^***^	0.249^***^	−0.141^***^	0.086^**^	0.018	0.066^*^	0.196^***^	−0.202^***^
Acceptance	0.170^***^		0.199^***^	0.078^**^	0.145^***^	0.018	0.139^***^	−0.012	0.014
Rumination	0.294^***^	0.283^***^		−0.060	0.193^***^	0.019	−0.014	0.299^***^	0.179^***^
Positive refocusing	−0.113^***^	0.075^**^	−0.041		0.146^***^	0.138^***^	0.118^***^	0.044	0.059^*^
Refocus on planning	0.090^**^	0.183^***^	0.171^***^	0.190^***^		0.351^***^	0.012	−0.130^***^	0.030
Positive reappraisal	0.025	0.029	0.022	0.231^***^	0.450^***^		0.488^***^	−0.116^***^	0.003
Putting into perspective	0.063^*^	0.159^***^	−0.011	0.140^***^	0.011	0.346^***^		0.026	0.009
Catastrophizing	0.226^***^	−0.016	0.293^***^	0.063	−0.144^***^	−0.100^***^	0.031		0.471^***^
Other-blame	−0.159^***^	0.013	0.119^***^	0.058^*^	0.023	0.002	0.008	0.321^***^
Arousal anxiety * sex	0.017	0.034	0.001	−0.054	0.008	−0.023	0.003	0.031	0.001
Apprehension anxiety * sex	0.029	−0.053	−0.013	0.001	−0.009	0.006	0.009	−0.006	0.011
Valence depression * sex	0.114^**^	0.005	−0.071	0.019	−0.026	0.022	−0.017	−0.001	0.114^*^
Anhedonic depression * sex	−0.105^*^	−0.033	0.089^*^	−0.068	0.113^*^	−0.012	−0.060	0.011	−0.099
*R*^2^ change	0.004	0.001	0.002	0.004	0.004	0.000	0.002	0.000	0.003
*F* change significance	0.040	0.665	0.336	0.133	0.039	0.965	0.301	0.860	0.200

*Interactions of the types of anxiety and depression with sex were entered in the second block. The variables in the interactions were centered at their means*.

* *p < 0.05*;

** *p < 0.01*;

*** *p < 0.001. p < 0.05 was used for hypotheses testing. All the other analyses used a corrected value of p < 0.001. Sex coding: 0 = male, 1 = female*.

**Table 6:** Results of regression analyses (standardized Betas) for the predicted aggregated adaptive and maladaptive cognitive emotion regulation strategies (CERS) with types of anxiety and depression, age, sex, and the aggregated maladaptive and adaptive CERS (respectively) as predictors (*N* = 1,346).

	**Adaptive strategies**	**Maladaptive strategies**
Arousal anxiety	0.008	0.025
Apprehension anxiety	−0.070	0.158^***^
Valence depression	−0.056	0.186^***^
Anhedonic depression	−0.422^***^	0.356^***^
Age	0.075^**^	0.001
Sex	0.059^*^	−0.022
Adaptive strategies		0.177^***^
Maladaptive strategies	0.212^***^	
*R*^2^	0.202	0.333
Model parameters	*F*_(7, 1338)_ = 49.747	*F*_(7, 1338)_ = 96.913

* *p < 0.05*;

** *p < 0.01*;

*** *p < 0.001*.

### Arousal anxiety

Arousal anxiety did not significantly predict any of the CERS, neither by itself or in interaction with sex. Also, this type did not predict the use of adaptive or maladaptive aggregated strategies.

### Apprehension anxiety

Apprehension anxiety significantly predicted the use of rumination and positive refocusing (as predicted, the latter one was an inverse relationship). It did not interact with sex. When aggregated strategies were analyzed, only the maladaptive ones were predicted by apprehension anxiety.

### Valence depression

Valence depression turned out to be a significant predictor of other-blame (positive relationship) and refocus on planning (negative). Additionally, a significant interaction indicated that the relationship between valence depression and self-blame is moderated by sex in such a way that valence depression is a significantly stronger predictor of self-blame in women than in men. When aggregated strategies were analyzed, only the maladaptive ones were predicted by valence depression.

### Anhedonic depression

Anhedonic depression significantly predicted the use of self-blame, rumination, positive refocusing, positive reappraisal, putting into perspective, and catastrophizing. The relationship with positive refocusing, positive reappraisal, and putting into perspective was positive and with the remaining three strategies—negative. The significant interactions additionally showed that anhedonic depression is a stronger predictor of self-blame in men than in women and a significant predictor of refocus on planning in women. This type was the only significant (and the strongest of all types) predictor of both the aggregated adaptive and maladaptive strategies.

## Discussion

In this study, we analyzed the relationships between types of anxiety and depression on one side and cognitive emotion regulation strategies on the other. We based our approach on the model developed by Fajkowska (2013, 2015), assuming that anxiety and depression are personality types built on two main criteria: structural complexity and functions (reactive or regulative) in stimulation processing that may be related to the typical emotion regulation strategies used by people representing these types. According to this view, the patterns of cognitive emotion regulation strategies use are the behavioral markers that are the reflection of the underlying structure and functions of the affective types.

Consistently with previous studies (Garnefski et al., 2002b; Kraaij et al., 2002; Martin and Dahlen, 2005; Garnefski and Kraaij, 2007; Aldao et al., 2010; Joormann and Gotlib, 2010), the relationships that we found were in the expected direction, i.e., the maladaptive strategies were related positively and the adaptive strategies negatively to the types of anxiety and depression, supporting the idea that emotion regulation might be one of the important aspects of these phenomena. In our study, acceptance was the only strategy that was not predicted by any of the types. This result suggests that acceptance should be interpreted with caution, especially since some studies show that it should be considered as a maladaptive strategy (Martin and Dahlen, 2005). Interestingly, arousal anxiety did not predict the use of any cognitive emotion regulation strategy. This result is in line with the assumption that it is primarily a reactive, “physiological” type, therefore weakly relating to personality characteristics of a regulative nature, such as emotion regulation strategies. On the other hand, anhedonic depression predicted the use of the largest number of strategies, including three adaptive (positive reappraisal, positive refocusing, and putting into perspective, all related negatively) and three non-adaptive (self-blame, catastrophizing, and rumination, all related positively). In the typology we used this type is the most structurally complex: positive reappraisal, positive refocusing, putting into perspective, and rumination might be related to emotional-motivational deficits and problems with attentional control, including inability to sustain attention on emotional material, lowered ability to sustain effort in processing emotional material, regardless of its valence, and problems with concentration of attention. Furthermore, the emotional-motivational deficits impair the ability to activate a goal of influencing the emotion-generative processes that is involved in the emotion regulation (Gross et al., 2011). Self-blame, on the other hand, is related to the very low positive affect and high negative affect that also constitute the structure of anhedonic depression. Additionally, anhedonic depression is a regulative type, which, according to our predictions, should be related to a more frequent use of the strategies in general. This finding can be interpreted in the light of the results of a recent study (Liu and Thompson, 2017) which suggest that patients diagnosed with depression are capable of using emotion regulation strategies, but their selection ability is impaired.

We hypothesized that regulative apprehension anxiety, with its dominating cognitive component (worrisome thoughts, reduced attentional control), will predict the more frequent use of rumination. The results confirmed this hypothesis. Regulative anhedonic depression (which is also characterized by reduced attentional control) predicted the use of rumination as well. Studies usually show that rumination is related to depression (Garnefski et al., 2002b, 2004; Martin and Dahlen, 2005; Garnefski and Kraaij, 2007; Joormann and Gotlib, 2010; Van Loey et al., 2014), but some also show its relationship with anxiety (Garnefski et al., 2002b; Martin and Dahlen, 2005; Garnefski and Kraaij, 2007). Rumination and worry, which are commonly associated with depression and anxiety, respectively, seem to have a lot in common: they are both repetitive, uncontrollable, and negative. These shared characteristics may be responsible for the overlap in the results. However, research shows that they differ in content, especially concerning the temporal orientation of the thoughts: in anxiety they mostly concern the future, while in depression—the past (Ehring and Watkins, 2008). Our approach might help understand the previously found effects by focusing on the structural and functional characteristics of the anxiety and depression types. Moreover, we expected that the reduced attentional control in anhedonic depression and apprehension anxiety will be related to a less frequent use of positive refocusing, which was confirmed by our data. Additionally, we predicted that anhedonic depression, because of its structural components, will be related to a more regular use of self-blame and catastrophizing. This hypothesis was also confirmed. The unique strategy predicted by valence depression was other-blame (which was related only to this type), in accordance with our assumptions. Refocus on planning and self-blame (in women) were also predicted by valence depression. Other- and self-blame are related to the Negative Affect component that includes anxiety, tension, hostility, and anger, while refocus on planning might be negatively related because valence depression is a reactive type, which is by definition characterized by more automatic than strategic processing (Fajkowska, 2017).

Concerning the functional role of the types of anxiety and depression, we previously reported that the regulative types correlated more strongly with the use of aggregated strategies than the reactive types (Fajkowska et al., 2018). In the current, more detailed analyses the data revealed that regulative anhedonic depression is related to a greater number of cognitive emotion regulation strategies than reactive valence depression. Anhedonic depression was the strongest predictor of the adaptive and maladaptive strategies after controlling for the other types of anxiety and depression. This result contradicts previous findings which suggest that adaptive emotion regulation strategies are less strongly related to psychopathology, including depression (Aldao and Nolen-Hoeksema, 2010). Similarly, regulative apprehension anxiety predicted a larger number of the strategies compared to arousal anxiety, which was not related to the strategies at all. Based on the data, we can conclude that the regulative types use a wider range of cognitive emotion regulation strategies than the reactive types, which confirms our predictions.

Previous results (Garnefski et al., 2004; Nolen-Hoeksema and Aldao, 2011; Martins et al., 2016) suggested that women tend to use rumination, positive refocusing, catastrophizing, reappraisal, and acceptance more, and self- and other-blame less often than men, but another study found extremely weak gender effects (Garnefski and Kraaij, 2006). Our data shows that in general women use more acceptance, rumination, and putting into perspective than men do. When anxiety, depression, and remaining strategies were controlled, the results showed that women use less self- and other-blame than men. However, these differences did not directly translate to relationships between the types of anxiety and depression and the CERS. The same strategies were predicted by apprehension anxiety in women and men, and arousal anxiety did not predict the use of any strategies in either women or men. However, valence depression was a stronger predictor of self-blame in women, and anhedonic depression—in men. Additionally, anhedonic depression was a stronger—and positive—predictor of refocus on planning in women. These results suggest that the mechanisms responsible for the occurrence and maintenance of depression types might manifest themselves in different ways in women and men. However, the moderating effects of sex should be replicated before any conclusions can be drawn, especially that neither reached the stricter significance level set by the multiple comparisons correction.

## Conclusions

To sum up, our study showed that the proposed types of anxiety and depression predict different patterns of the cognitive emotion regulation strategies use, and that these strategies are related to the proposed structural and functional characteristic of the types. We controlled for the effects of co-occurrence, as it allows to study the effects and characteristics of “pure” types of anxiety and depression, which enables us to better understand their mechanisms. Age did not turn out to be a significant predictor of any of the strategies. The contribution of the affective types to the prediction of the cognitive emotion regulation strategies use is small to moderate, which is not surprising, given that the affective types can be characterized by various behavioral markers. This issue needs to be addressed in future studies. For example, so-called overt emotion regulation strategies, such as drinking alcohol, eating, or seeking advice have been shown to be related to anxiety and depression symptoms (Aldao and Dixon-Gordon, 2014). Similarly, there is some evidence that positive emotion regulation is also impaired in anxiety and depression (Carl et al., 2013). Another factor that might play a role in the relationship between anxiety and depression and the cognitive emotion regulation strategies is the context of the emotions occurrence. A greater flexibility in the use of strategies was shown to be related to better mental health (Aldao and Nolen-Hoeksema, 2012). The main limitation of the study is its self-report character. Moreover, even though the underlying theory assumes that the strategies use is the result of the anxiety and depression, the direct causality cannot be confirmed in this study and needs further research.

Nevertheless, these results further validate the proposed classification of anxiety and depression, and at the same time allow for a better understanding of their underlying mechanisms. Research suggests that the use of cognitive emotion regulation strategies influences emotional responding (for a review see: Cisler and Olatunji, 2012), which in turn might be related to the maintenance of symptoms of anxiety and depression. Therefore, our findings might turn out to be useful in clinical practice. For example, both regulative types are related to rumination and problems with positive refocusing, which points to deficits in cognitive control that can be targeted in therapy. The negative relationships of anhedonic depression with adaptive strategies additionally suggest that therapy should focus on both teaching to stop using the maladaptive strategies and at the same time to use the adaptive ones, especially in this particular type. Moreover, our results suggest that considering CERS may be helpful in distinguishing types of anxiety and depression.

Interestingly, according to our results, types of anxiety are related to a less frequent use of the CERS than types of depression. Theoretically, it seems that cognitive regulation of emotion is less mentally available to anxious than depressive individuals. For some reasons they might not be able to use them. Although the latter ones rather use maladaptive and ineffective cognitive strategies of emotion regulation, it still suggests that cognitive control over emotional states seems to be crucial in depression. Therefore, the transformation from negative to positive strategies should be recommended as very important in the therapeutic protocol of curing depression. However, the question arises about what kinds of strategies are more available/typical to anxious people. Behavioral strategies are one possible answer. These issues can be the focus of further studies and speculations related to therapy of anxiety (e.g., how to regulate the level of anxiety: by building adaptive cognitive strategies of emotion regulation or by incorporating behavioral ones?).

The gender differences should also be considered in therapy, as our results show possible, however small, differences between men and women in typical patterns of CERS use in types of depression. Therefore, a greater focus on strategies typical for each gender could turn out a useful approach. However, research on clinical samples might bring different effects, as the present study was conducted on a general population.

## Data availability

The dataset generated for this study can be found in the Mendeley Data repository (https://data.mendeley.com/datasets/r45ht8hrnb/1).

## Author contributions

MF and ED: contributed to the study design and supervised data collection; ED: analyzed the data and drafted the manuscript; MF: provided critical revisions; ED and MF: approved the final version of the manuscript.

### Conflict of interest statement

The authors declare that the research was conducted in the absence of any commercial or financial relationships that could be construed as a potential conflict of interest.
